# Short report: The role of oral hypersensitivity in feeding behaviors of young autistic children

**DOI:** 10.1177/13623613221135091

**Published:** 2023-02-24

**Authors:** Kelsey Thompson, Anna Wallisch, Sallie Nowell, Jessica Meredith, Brian Boyd

**Affiliations:** 1 The University of North Carolina at Chapel Hill, USA; 2 The University of Kansas, USA

**Keywords:** autism spectrum disorders, nutrition/feeding, pre-school children, sensory impairments

## Abstract

Feeding problems are common among autistic children and are linked to negative health consequences. Therefore, understanding feeding problems and factors that influence these behaviors is important for developing supports for children and families. While certain sensory processing patterns are commonly associated with feeding problems, less is known about the link between sensory processing and feeding behaviors in autism, as well as how parent behaviors and feelings during mealtime differ based on child sensory preferences. This research examined two groups of young autistic children who were reported to be picky eaters by their parents: those with and those without oral hypersensitivity. Children with oral hypersensitivity had more difficulty with food acceptance, and their parents reported more negative feelings around feeding their child. However, the two groups of children (oral hypersensitive and not) did not differ in their medical/oral motor symptoms, mealtime behavior, or parent use of strategies at mealtimes. This research supports the need for personalized treatment strategies based on the child’s sensory preferences to support both the child and parent in managing mealtimes.

**Lay abstract:**

Feeding problems are common among autistic children and are linked to negative health consequences. Therefore, understanding feeding problems and factors that influence these behaviors is important for developing supports for children and families. While certain sensory processing patterns are commonly associated with feeding problems, less is known about the link between sensory processing and feeding behaviors in autism, as well as how parent behaviors and feelings during mealtime differ based on child sensory preferences. This research examined two groups of young autistic children who were reported to be picky eaters by their parents: those with and those without oral hypersensitivity. Children with oral hypersensitivity had more difficulty with food acceptance and their parents reported more negative feelings around feeding their child. However, the two groups of children (oral hypersensitive and not) did not differ in their medical/oral motor symptoms, mealtime behavior, or parent use of strategies at mealtimes. This research supports the need for personalized treatment strategies based on the child’s sensory preferences to support both the child and parent in managing mealtimes.

## Introduction

Feeding problems are common among autistic children (Leader et al., 2020). While “feeding problems” is a broad term, autistic children most frequently demonstrate food selectivity or refusal, and may also have challenges with oral motor skills, gastrointestinal conditions, and difficult mealtime behaviors (Crasta et al., 2014; Lane et al., 2014; Leader et al., 2020). These feeding problems are often linked to poor health consequences including nutritional deficiencies and obesity (e.g. Leader et al., 2020) and cause significant parental stress (e.g. Nadon et al., 2011).

Multiple mechanisms are thought to be linked to feeding problems in autistic children including repetitive behaviors, sensory processing, co-occurring health conditions, and difficulties with the social aspects of mealtime (e.g. Leader et al., 2020; Nadon et al., 2011). Despite the many potential mechanisms implicated, it remains unclear the degree to which they are linked to feeding problems. Understanding which mechanisms underlie specific feeding behaviors could have significant clinical implications, such as tailoring the treatment approach based on the child’s behavior.

One mechanism with particularly strong evidence is sensory processing. Sensory processing differences are common in autism (Robertson & Baron-Cohen, 2017), and multiple studies have linked oral hypersensitivity to feeding challenges in autistic children (e.g. Kral et al., 2015; Shmaya et al., 2017). However, these studies have addressed wide age ranges, potentially limiting the applicability of the findings, and most have yet to examine the role of parent behaviors during mealtime.

There is a known relationship between parent and child behaviors in the presence of mealtime challenges and sensory processing preferences. For example, sensory processing differences can contribute to caregiver stress (Nieto et al., 2017; Schaaf et al., 2011). In addition, caregiver behavior during mealtimes can impact children’s eating behaviors (e.g. pressure from parents is linked to picky or problem eating in children; see Chilman et al., 2021). Despite both sensory processing differences and feeding problems impacting caregivers, there is little understanding of how these two domains relate and interact among parents of autistic children during mealtimes.

It is important to understand how specific types of eating behaviors are linked to patterns of oral sensitivity and parent mealtime behaviors so that interventions can be tailored to individual child and parent needs. In addition, by examining eating behaviors during early childhood, we may begin to understand ways to prevent the associated negative health consequences. Therefore, the purpose of this study was to determine differences in eating behaviors in young autistic children based on oral sensory processing patterns.

## Methods

### Participants

Parents of children aged 3–6 years with a diagnosis of autism were recruited from a university diagnostic center registry and from social media postings. We included this age range because picky eating is common and developmentally appropriate until age 2 (Emond et al., 2010) and feeding behaviors of autistic children often diverge from non-autistic children at this age. Children were included based on age, autism diagnosis, and if the caregiver reported their child as a “picky eater.” All caregivers e-consented to participating, and this study was approved by the University of Kansas Medical Center Institutional Review Board.

Seventy-nine caregivers in the United States initiated the REDCap survey; however, only caregivers who completed the measures critical to our research question were included (*N* = 68). Six caregivers reported their child’s age as older than 6 years 11 months and were excluded, leaving a total sample of 62 caregivers. Caregivers who completed the survey were mostly White, non-Hispanic, and female identifying. Children in the study were mostly male (60%) with an average age of 56.27 months (*SD* = 12.06). See Table 1.

**Table 1.** Participant demographics.

	Oral hypersensitive *n* = 34		Oral non-sensitive *n* = 28	
	*M* (*SD*)	% (*n*)	*M* (*SD*)	% (*n*)
Child age (months)	55.68 (12.53)	–	57.00 (11.65)	–
Child sex (female)^a^	–	38.2 (13)	–	35.7 (10)
Child below average cognitive ability	–	44.1 (15)		14.3 (4)
Caregiver sex (female)	–	94.1 (32)	–	100 (28)
Caregiver role				
Mother	–	94.1 (32)	–	96.4 (27)
Father		2.9 (1)	–	0
Grandparent		2.9 (1)	–	3.6 (1)
Caregiver race^b^				
Black	–	8.8 (2)	–	10.7 (3)
White	–	91.2 (29)	–	71.4 (20)
Bi-racial / multi-racial	–	5.9 (2)	–	10.7 (3)
Asian	–	2.9 (1)	–	3.6 (1)
Caregiver ethnicity (Latino)	–	7.5 (4)	–	3.6 (1)

*SD*: standard deviation.

a Data missing from one child in oral hypersensitivity group.

b Data missing from one child in oral non-sensitive group.

### Procedure

Caregivers of autistic children were asked to complete an online REDCap survey about mealtime and oral sensory processing patterns. Community members were not involved in the development or dissemination of this study.

### Measures

Demographic information was collected via an author-created survey and gathered information about the caregiver completing the survey and their autistic child (see Table 1).

#### Sensory Profile–2 Oral Processing subscale

Caregivers completed the Sensory Profile–2 Oral Processing subscale (SP-2; Dunn, 2014), which uses a 5-point Likert-type scale (i.e. 1 = *almost never* to 5 = *almost always*). The Oral Processing subscale has 10 items; children were grouped into “oral hypersensitivity” (oral-HYPER) or “oral non-sensitive” (oral-NON) groups based on scores on the five Sensitivity quadrant items, which relate to how much the child is detecting a specific sensory input (Dunn, 2014). In general, for the Oral Processing subscale, the Sensitivity items ask about taste / smell sensitivity (2 items), texture sensitivity (2 items), and biting tongue or lips (1 item). When children show “less than or much less than others” scores, they are at least 1 standard deviation (*SD*) below the normative sample and demonstrate *decreased* (or hypo) responses to sensory stimuli. When children score in the “more than or much more than others” category (1 *SD* above the normative sample), they are showing *increased* (or hyper) responses in that sensory domain. An oral hypersensitivity score was calculated based on previous work (Wallisch et al., 2022), and the SP-2 normative data, whereby children with an average response score above 2.4 on the sensitivity oral processing questions were considered oral-HYPER. This resulted in 34 children in the oral-HYPER group and 28 children in the oral-NON group. No children in the sample scored in the *hypo*sensitive range on this scale.

#### Behavioral Pediatric Feeding Assessment Scale

The Behavioral Pediatric Feeding Assessment Scale (BPFAS) is a widely used, parent-report measure of feeding problems in children (Crist & Napier-Phillips, 2001), and is frequently used to assess autistic children (Allen et al., 2015). The 35 items of the BPFAS are divided into two subscales: 25 items related to child eating behaviors (e.g. “will try new food,”) and 10 items related to caregiver Feelings (e.g. frustration) and Problematic Strategies (e.g. force feeding) during mealtime. Each behavior is rated by frequency from 1 (*never*) to 5 (*always*) and caregivers indicate if the behavior is problematic for them (*yes* or *no*). The mean frequency score, as well as subscale frequency scores (Food Acceptance, Medical/Oral Motor, and Mealtime Behavior) were calculated as described in Allen et al. (2015) for the child feeding questions. The parent items were divided into the two subscales for analysis: Feelings (items 26, 29, 30, 34, and 35) and Problematic Strategies (items 27, 28, 31, and 32). Item 33 “I disagree with other adults (example, my spouse, the child’s grandparents) about how to feed my child” was excluded from analyses as it did not fit into either construct.

### Data analysis

First, chi-square or *t*-test statistics, depending on variable type, were used to examine differences in child sex and age, as well as a parent-reported cognitive ability across groups to determine any covariates for the model. Cognitive ability was assessed by the following question: “Indicate your child’s current level of IQ/functioning,” with three responses (below average IQ, average IQ, above average IQ). For our purposes, cognitive ability was transformed to a binary variable (i.e. IQ below average or IQ average / above average) as a proxy to account for an intellectual disability. Then, a multivariate analysis of covariance (MANCOVA) was completed with child’s group (i.e. oral-HYPER or oral-NON) as the independent variable and mean scores on the three BPFAS child subscales (Food Acceptance, Medical / Oral Motor, and Mealtime Behavior), and the two BPFAS caregiver subscales (Feelings, Problematic Strategies) as dependent variables.

## Results

When examining differences between groups, results indicated there were no significant group differences on child sex (χ^2^ *=* 0.09, *p* = 0.77) or age, *t*(60) = 0.43, *p* = 0.67; however, there were significant differences in cognitive ability (χ^2^ = 6.43, *p* = 0.01). Therefore, we added cognitive ability as a covariate in our model. Overall, MANCOVA results suggested a significant model, *F*(5, 55) = 4.41, *p* = 0.002, Wilks’s Λ = 0.71, $\eta_{p}^{2}$ = 0.29.

When examining group comparisons on the dependent variables, children with oral-HYPER (*M* = 3.49, *SD* = 0.53) compared to the oral-NON group (*M* = 3.07, *SD* = 0.53) had significantly higher scores on the Food Acceptance subscale of the BPFAS indicating more feeding acceptance problems, *F*(1, 59) = 6.54, *p* = 0.01, $\eta_{p}^{2}$ = 0.10. Furthermore, the parent subscales of the BPFAS indicated parents of children in the oral-HYPER group experienced more negative feelings around feeding their child compared to the parents of children in the oral-NON group, *F*(1, 59) = 7.65, *p* = 0.01, $\eta_{p}^{2}$ = 0.12. There were no statistically significant differences between the two groups on the Medical / Oral Motor and Mealtime Behavior or the Parent Strategies subscales of the BPFAS (see Table 2).

**Table 2.** Group differences in BPFAS scores.

BPFAS subscales	Oral hypersensitive *n* = 34	Oral non-sensitive *n* = 28	*F* value	*p* value
	*M* (*SD*)	*M* (*SD*)		
Child				
Food Acceptance	3.49 (0.53)	3.07 (0.53)	6.54	0.01
Medical / Oral Motor	2.07 (0.54)	1.81 (0.39)	2.63	0.11
Mealtime Behaviors	2.72 (0.56)	2.74 (0.82)	0.31	0.58
Caregiver				
Feelings	2.73 (0.67)	2.14 (0.65)	7.65	0.01
Problematic Strategies	2.60 (0.58)	2.46 (0.51)	0.31	0.58

BPFAS: Behavioral Pediatric Feeding Assessment Scale; *SD*: standard deviation.

a Caregiver report cognitive ability was included as a covariate.

## Discussion

We examined how feeding problems differ in autistic children with and without oral hypersensitivity. In our sample, children with oral hypersensitivity had significantly more difficulties with food acceptance, and caregivers reported significantly more negative feelings related to child feeding compared to children without oral hypersensitivity. However, the groups did not differ in terms of their mealtime behavior nor their medical and oral motor feeding problem symptoms. They also did not differ in terms of caregiver problematic strategies during feeding.

Children with oral hypersensitivity were more selective about trying new foods and eating foods from a variety of food groups. These findings are consistent with prior research that suggests oral hypersensitivity is associated with selective eating, both among neurotypical and neurodiverse children (e.g. Shmaya et al., 2017; Zickgraf et al., 2020). In addition, oral or taste/smell hypersensitivity has been associated with food refusal and limited variety in autistic children, all consistent with our findings on the BPFAS (Kral et al., 2015; Lane et al., 2014). Interestingly, our sample does not support a connection between oral hypersensitivity and mealtime behavior. This is in contrast to some prior work which has found associations between taste/smell sensitivity and mealtime behavior in autistic children (e.g. Crasta et al., 2014; Marshall et al., 2016). This unexpected finding may indicate that mealtime behaviors are more similar across autistic children, regardless of sensory preferences. Alternatively, this may reflect the usage of different measures of mealtime behaviors across studies. For example, this study utilized the BPFAS, whereas most other studies reporting the connection between oral sensory processing and mealtime behavior utilized the Brief Autism Mealtime Behavior Inventory to measure mealtime behaviors (for review, see Page et al., 2022). Finally, it is plausible that differences in the characteristics of our sample (e.g. child age, cognitive level) compared to other studies may also contribute to contrary findings; however, a strength of our study is the narrow age range. We controlled for cognitive ability due to group differences, and prior research is mixed in terms of the connection between feeding problems and cognitive skills in autism (e.g. Allen et al., 2015; Page et al., 2022). Furthermore, research on the link between sensory processing and cognitive ability is also mixed in autism (for review, see Dunn et al., 2016) with some studies suggesting a link between sensory processing and cognitive abilities (e.g. Zachor & Ben-Itzchak, 2014), other studies finding no link (e.g. Nadon et al., 2011; O’Donnell et al., 2012), and others finding that cognitive abilities moderate the relationship between sensory processing and challenging behaviors (Werkman et al., 2020). Future research should investigate the interaction between cognitive ability or executive functions and eating behaviors in autism.

Overall, our findings reinforce that oral hyperresponsivity likely impacts the food acceptance of autistic children. Future research should continue investigating our contrary findings related to mealtime behaviors as well as other mechanisms to understand the heterogeneity of mealtime behaviors in autism. While long-standing research in pediatric obesity has indicated the bidirectional effect between child feeding behaviors and parent feeding strategies, autism feeding research has primarily focused on child feeding behaviors. Interestingly, our study revealed that caregivers of children with oral hypersensitivity experienced more negative emotions around feeding their child but did not differ from caregivers of children without oral hypersensitivity in terms of strategies used during mealtimes. The group differences in experiencing negative emotions are consistent with prior work (Nieto et al., 2017; Schaaf et al., 2011). The null findings regarding differences in parent strategies may reflect the relatively extreme strategies included on the BPFAS (i.e. force feeding, using threats) or may mean that parents of autistic children use similar strategies regardless of their child’s sensory processing. Future studies should focus on identifying commonly used parent strategies, their effectiveness, and their association with child characteristics.

## Limitations and future directions

While our sample did not include children with oral *hypo*sensitivity, future studies should examine how these patterns are related to feeding behaviors among autistic children. Our survey did not ask parents to report if their child had any co-occurring conditions, and it is unclear if other conditions contributed to our results. Furthermore, our sample size was small, which limited our ability to detect differences of moderate and small effect size; however, this sample reflects a tight age range designed to detect feeding problems specific to preschoolers. We assessed cognitive ability through a parent-report item, future studies should report validated assessments of cognitive ability to further elucidate any connections between mealtime behaviors and cognition. Finally, the lack of racial and ethnic diversity in our sample reduces the generalizability of our findings. More research is needed to understand cross-cultural considerations of mealtime and eating behavior.

Overall, this study concludes that autistic children with oral hypersensitivity experience greater difficulties with food acceptance than autistic children without oral hypersensitivity, consistent with prior work. However, in contrast to prior research, the oral hypersensitive group did not exhibit differences in mealtime behavior or medical/oral motor skills. Given these findings, autistic children with oral hypersensitivity may benefit from early feeding intervention focused on sensory preferences to improve food acceptance and prevent long-term health implications. Finally, parents of children with oral hypersensitivity experienced more negative emotions around mealtime, therefore, future research should work to elucidate effective parent strategies for supporting children with sensory processing differences.
